# Virtual reality boxing: impact of gaze-contingent blur on elite boxers performance and gaze behavior

**DOI:** 10.3389/fspor.2024.1430719

**Published:** 2024-12-24

**Authors:** Annabelle Limballe, Richard Kulpa, Eulalie Verhulst, Simon Ledouit, Simon J. Bennett

**Affiliations:** ^1^ Univ Rennes, Inria, M2S, Rennes, France; ^2^ School of Sport and Exercise Sciences, Liverpool John Moores University, Liverpool, United Kingdom

**Keywords:** blur, sport, boxing, anticipation, virtual reality

## Abstract

It is essential in combat sports such as boxing for athletes to perceive the relevant visual information that enables them to anticipate and respond to their opponent's attacking and defensive moves. Here, we used virtual reality (VR), which enables standardization and reproducibility while maintaining perception-action coupling, to assess the influence of a gaze-contingent blur on the visual processes that underpin these boxing behaviours. Eleven elite French boxers were placed in an immersive and adaptive first-person VR environment where they had to avoid by dodging one or two punches, and then counterattack to strike their opponent. The VR boxing task was performed in a central blur, peripheral blur and control condition. The results showed that elite boxers outcome performance was resilient to blur, irrespective of its location in the visual field. However, there was an effect of blur on the eye gaze data, with participants spending less time looking at the left hand and plexus, and more time looking at the head and areas other than the boxer, in the peripheral blur condition. Overall, then, our study contributes to growing evidence that performance in dynamic interceptive sports can be maintained when the visual stimulus is artificially blurred. In future work, it will be relevant to consider whether VR training with a gaze-contingent blur can facilitate learning, transfer, and/or reintroduction after injury, to a real boxing situation.

## 1 Introduction

Boxing is a combat sport characterized by very strong spatio-temporal and event constraints (1, 2). The speed of the opponent's attacking punches coupled with the short participant-opponent distance, mean that anticipation is key to successful performance (2, 3). Contributing to this visual-cognitive skill is likely to be the use of low spatial frequency visual information from the opponent (i.e., motion), perceived through peripheral vision (3, 4). Indeed, gaze location tends to be around the trunk or the head of the opponent in order to facilitate perception of information from the other peripheral locations such as the opponent's fists (5, 6). To date, however, work in combat sports has studied interactions with real opponents, which are difficult to control and manipulate, or 2D screen projections that do not preserve a realistic perception-action coupling (7, 8). To overcome such limitations, the current study considered the potential of virtual reality (VR) to provide an immersive and adaptive environment (9, 10) in which boxers can practice without being exposed to the risk of osteoarticular injury (e.g., fists), as well as repeated blows to the head and the associated potential for mild traumatic brain injury (11, 12). In addition to providing a realistic simulation of the real-world task, VR enables targeted manipulations of the visual environment and thus the information available. Therefore, VR offers great promise in improving upon the traditional spatial occlusion (i.e., specific parts of the stimulus are removed) and temporal occlusion (i.e., the entire stimulus is removed at specific times relative to a key event) paradigms that have been used frequently to determine what and when visual information is perceived in sport settings [for a review see (13)].

Motivated by lab-based tests of anticipation in tennis (14) and badminton (15), and decision making in basketball (16) and soccer refereeing (17), Limballe et al. (18), used virtual reality to investigate the effect of a gaze-contingent Gaussian blur in boxing. The logic was that by minimizing access to high spatial frequency input from a specific part of the visual stimulus (i.e., contingent upon gaze), attention would be directed to potentially more relevant low spatial frequency input such as motion and form (16, 19). Importantly, VR was used to overcome limitations of the previous studies associated with recording of button/verbal responses to manipulations of blur on a 2D video display. Such methods decouple the mutuality between perception and action (i.e., no motor response and no access to binocular depth information), which is a crucial component of combat sports (7). Consistent with previous studies, it was found that the ability to intercept an attacking punch was relatively impervious to the presence of blur whether it be located in central or peripheral vision. However, despite the VR environment offering an immersive and adaptative first-person scenario, the chasing parry-like task may not have been sufficiently representative of boxing because it did not require participants to dodge or take a punch in the guard position (20). With a punch duration typically shorter than 200 ms, and a similar reaction time (21), being able to anticipate and dodge an incoming punch requires the pick-up of early information, likely from the opponent's body movement before the onset of the punch. Having dodged the opponent's punch, the boxer then has the opportunity to counterattack and land decisive blows that can help them win the fight.

Given the importance of representative design and realism of the experimental task (22), the current study examined the impact of a gaze-contingent blur during an immersive VR boxing task, which required a counterattack after dodging an attacking punch from their opponent. We studied elite boxers with the expectation that they would have sufficient experience and skill to respond appropriately to the first (dodge) and/or second (counterattack) phase of the boxing task. We also determined the distance between the participant and opponent, which is crucial in combat sports for controlling the opportunity to attack or defend, and thus to gain advantage over the opponent (23). In addition, to determine if the blur manipulation affected gaze location, and thus the potential to perceive information from different parts of the visual field, we calculated the spatial distribution of gaze location, as well as the percentage of time that gaze was located within specific areas of interest (AOIs). Consistent with previous work (14, 15, 18), we chose to implement a Gaussian blur rather than a dioptric blur (24, 25). The latter is equivalent to optical defocus caused by some visual impairments (e.g., myopia or inaccurate accommodation), and has been used to help improve understanding of visual impairment classifications for Paralympic sport (26). However, as discussed by Strasburger et al. (27), high spatial frequencies can still be detected under dioptric blur (e.g., orientation of a sine-wave grating) due to spurious resolution, as can differences between blurred optotypes with practice. A gaze-contingent Gaussian blur that updates on each sample of gaze data does come with a computational burden, and does not have a simple relationship with visual acuity, but it does avoid spurious resolution that could impact upon perception of high spatial frequencies.

## 2 Materials and methods

### 2.1 Participants

Eleven male participants with a mean age of 23 years (±2.44 years) completed the experiment. They were elite athletes from French boxing federation, who competed at national to international level; Tier 4 of McKay et al. (28), Participant Classification Framework. All participants were healthy, without injuries, right-handed and had normal or corrected-to-normal vision. They signed a written consent form and were free to withdraw from the experiment at any time. The experimental protocol was made in respect of the Declaration of Helsinki and accepted by the national ethical committee (CERSTAPS IRB00012476-2022-31-10-202). No personally-identifiable information (e.g., individual participant's years of practice and/or career track record) could be made available.

### 2.2 Task and stimuli

The set-up was adapted from Limballe et al. (18). The experiment took place in a real boxing ring (Olympic size, 6.10 m × 6.10 m inside the ropes) in the French boxing federation training center. Participants were immersed in the virtual environment using an HTC VIVE EYE PRO (110 deg FOV; 2160*1200 pixel image resolution; 0.011s temporal resolution), with HTC VIVE trackers attached to each wrist. Eye movements were recorded with the integrated eye tracking system (1 deg spatial resolution, 60 Hz temporal resolution) and used to control the gaze-contingency manipulation. Gaze location in 3D VR-centered coordinates was calculated by combining the 3D position of the head and the 3D cyclopean gaze vector. Calibration of gaze location within the virtual environment for each participant was done prior to each block of 60 trials using the HTC VIVE EYE PRO calibration tool. The VR environment was built in Unity (v. 2020.3.29f1) and presented a virtual opponent within a virtual boxing ring of official size (6.1 m × 6.1 m in Olympic boxing) that matched the size of the real ring in which participants completed the experiment. Realism was increased by the inclusion of ropes in the physical and virtual boxing rings. Participants were free to move within this ring but were also constrained by the ropes.

**Figure 1:**
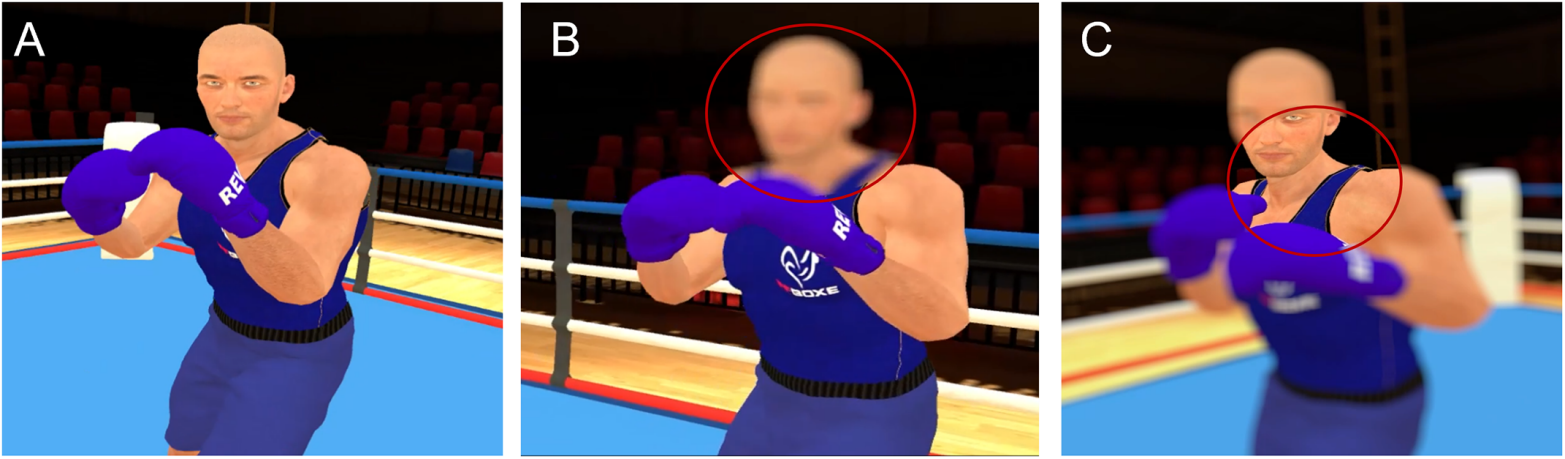
Representation of the viewing conditions. **(A)** control, **(B)** central blur, **(C)** peripheral blur. VR user view. For emphasis, an outline has been overlaid on the gaze- contingent window in panel **(B,C)**, but this was not visible to participants.

To provide realistic 3D coordinate data to animate the virtual opponent, motion capture was conducted on two high level boxers (one male, one female). They were both right-handed and had an orthodox stance in which they lead with the left hand and left foot. The resulting virtual opponent was a male avatar, whose proportions were not affected by the real boxers' characteristics. The virtual opponent was resized according to the participant size in order to ensure that the view of the virtual opponent within the participant visual field was similar and that the virtual opponent launched punches to similar body target (head or body). Participants faced the virtual male boxer (see Figure 1) in the virtual boxing ring. The virtual boxer was initially shown in a guard position 1 m away from the participant and maintained a face-to-face orientation by rotating according to the participant's movement. The virtual opponent moved toward the participant and launched an attacking sequence when he was at punching distance. This distance was pre-determined and adapted to each sequence based on punch extension to make sure that every punch touched the participant if they didn't move. For each sequence, punch extension was calculated as the distance between the virtual opponent's fist at the start and end of the punch (from pre-experimental motion capture) using the root position in Unity. As a result, a hook punch that had shorter punching distance than a straight punch would have been launched when the virtual opponent was closer to the participant. Before the experiment, participants were informed that they could withdraw whenever they wanted, and that they should inform the experimenter if they experienced symptoms of cybersickness. We also limited the total time of the experiment in VR in order to help prevent cybersickness, and gave participants a quick familiarization phase where they explored the virtual ring (30 s) and faced 10 attacking sequences (<60 s) before the start of the experiment. Participants started the round (i.e., a block of trials) when they were ready by punching the starting sphere in the scene (a red sphere located in front of them to trigger the onset of the block; then, the sphere disappeared).

The virtual opponent then launched successive attacks (i.e., trials), comprising 20 sequences (for details see Supplementary Table S1). There were 10 sequences of one punch (5 starting with left front hand, 5 with right back hand) and 10 sequences of two punches (4 starting with left front hand, 6 with right back hand). Participants were instructed to dodge the incoming punches and counterattack to hit the virtual opponent with their virtual hand. Auditory feedback indicated to the participant if they had been hit and/or hit the opponent. A successful dodge scored 1 point, whereas a successful dodge followed by a successful counterattack scored 2 points. No other responses, such as throwing a punch before or after the attack, were allowed or scored. This was done because the task required participants to anticipate in defence of an attacking sequence (i.e., dodge and counterattack), and not launch their own attacking attempt before the punch sequence began, or after the punch sequence ended. If the participant was touched by any punch in the sequence, it was considered as a failure and received a score of 0 points. After a sequence, the virtual opponent moved backward then started a new sequence was he was at punch distance. The 20 attacking sequences were presented in random order, 3 times per block (60 trials per block). As well as providing a realistic simulation of boxing, the sequence randomization minimized the opportunity for participants to learn the upcoming punches and respond without the need to attend to relevant stimuli. For example, a straight left punch to the head could have been a single punch, or followed by a straight right to the head or a right cross to the head, with each sequence requiring a specific response to achieve a maximum performance outcome. Each participant completed three blocks of 60 trials that were presented in a random-blocked order separated by a 2 min break. In each block, participants performed the virtual boxing task in one of 3 Viewing Conditions. In the control condition, participants were presented with normal vision of the opponent and surrounds. For the other 2 Viewing Conditions, a gaze-contingent blur manipulation was made relative to gaze location (for more detail see below and Figure 1), such that participants were presented with a peripheral blur or a central blur. In sum, participants completed 180 trials, which took approximately 10–15 min. We did not use power calculations to help determine the number of trials that represent a stable within-participant performance. Instead, our choice of 30 trials per unique combination of the 3 Viewing Conditions and 2 Sequence Types was based on previous research on blur in interceptive sporting tasks [Jackson et al. (14): *n* = 8; Mann et al. (24, 25): *n* = 15; Limballe et al. (18): *n* = 8 trials], while also taking into consideration the duration for which participants would be immersed in the VR setting. After the experiment, a debrief was performed in which participants and the coach were provided with an output performance report, an explanation of the study, and an opportunity to ask questions and comment on their subjective experience of the VR environment (e.g., feeling of presence and realism). They were also asked to report if they felt dizzy on a 0–10 scale. None of them experienced cybersickness or felt uncomfortable wearing the headset.

### 2.3 Gaze-contingency manipulation

The blur gaze-contingency manipulation was the same as in Limballe et al. (18). However, as we previously found no effect of level of blur, here only the medium level of blur (*σ* = 500, kernel size = 19) was used. A 5 deg blurred circle around gaze location was used to create the central blur condition, whereas in the peripheral blur condition, the blur was only applied to stimuli outside of this 5 deg circle (see Figure 1). The control condition did not include any blur manipulation. A Gaussian blur, which is a 2D-convolution operator, was used to manipulate the display. It took two inputs: the image of the participant's viewpoint generated at each frame and the Gaussian kernel whose size defines the blur intensity. A Gaussian function G has the following form:

$$G(x)=\frac{1}{2\pi \sigma^{2}}e^{-({\left(x\right)}^{2}/2\sigma^{2})}$$

where *σ* is the standard deviation of the distribution, *x* is the distance from its centre.

This Gaussian was applied to each pixel of the rendered image that was to be blurred. It took into account the neighbouring pixels with a weighting that is inversely proportional to its position (*x,y*) to the centre. The degree of blurring is thus determined by the standard deviation (*σ*) of the Gaussian, such that a stronger blur is obtained with a larger convolution kernel. It is important to note that Gaussian blur does not introduce noise in the image but instead simply acts as a low pass filter that makes transitions and edges between stimuli less prominent [for more details, see (29)].

### 2.4 Data analysis

**Figure 2:**
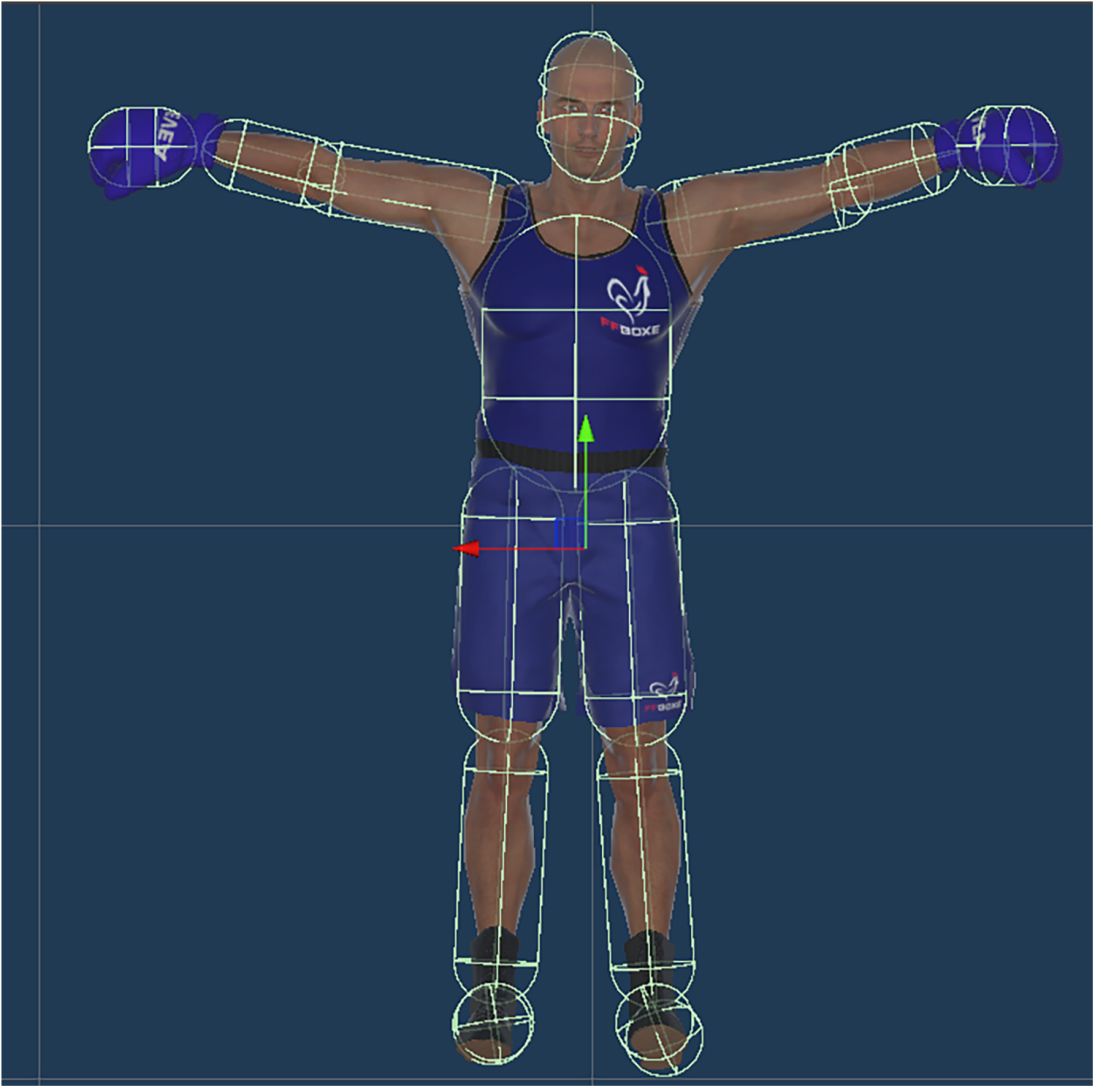
Representation of collider capsule placement on the 16 body parts of the virtual opponent.

The frame-by-frame distance between the participant and virtual opponent was calculated based on their respective head positions. The collider engine from Unity was used to detect a collision between the participant and the opponent's gloves (dodging), and the participant's hand and the opponent's body (counterattack). Having replaced missing gaze (8% of all raw data, including the rest time between the blocks) data using linear interpolation with the function*. interpolate()* from Panda Dataframe package in python, the global distribution of 3D gaze location in the VR environment was determined by calculating the volume of a 3D ellipse (i.e., ellipsoid) that contained 95% of the gaze data (i.e., excluding extreme data which could be outliers). Next, collider capsules were placed on the 16 body parts [see also (5)] of the virtual opponent using a dedicated software development kit (SDK) in the Unity software (see Figure 2). The GazeFocusChanged function in the IGazeFocusable interface from the software development kit's application programming interface (SDK's API) was used to determine the time a participant spent with their gaze located in each of the 16 AOIs, which were of a size that matched the moving body part. The time spent with gaze located at areas other than the opponent was calculated a posteriori by subtracting the sum time of gaze located on the virtual opponent from the total time of the block, and was considered as the 17th AOI. These data were expressed as the percentage time with gaze located at each of the 17 AOIs, and did not take account of whether gaze was stationary or moving due to eye and/or head movement.

### 2.5 Statistics

The primary interest was to examine if participants' outcome performance (dodge and/or counterattack), the average distance between the participant and opponent, and gaze location data was influenced by the inclusion of blur in the peripheral and central conditions compared to the control condition (Viewing Condition). It was also of interest examine if the influence of blur was mediated by the inclusion of one or two punches within an attacking sequence (Sequence Type). Accordingly, performance and gaze location data were analysed from three repetitions of the ten unique one or two punch sequences in each viewing condition.

To account for the crossed design and individual-participant variability in response to the experimental manipulations, all data was analysed using linear mixed effects modelling (lme4 and ordinal package in RStudio, v 2023.12). Outcome performance data (ordinal multinomial, ranked 0, 1 or 2 for each trial) was modelled using a cumulative link mixed model (clmm function from ordinal package, v. 2022.11–16). Participant-opponent distance was modelled according to a normal distribution with an identity link function. A top-down strategy was used in which all fixed effects were first included (e.g., main and interaction terms), as well as a random intercept and slope (*y* = Viewing Condition*Sequence Type + (1 + Viewing Condition| Participant). Fixed effects were sequentially removed based on their statistical significance determined using Wald Chi Squared tests (CAR package in *R*), and provisionally retained if they returned *p*-values of 0.1 or less. The reduced-effects model was compared to the full-effects model with conditional *R*^2^ (MUMIn package) and AIC. It was also compared to a reduced model with the same fixed effects but only a random intercept. In the final reduced model, only fixed effects at *p* ≤ 0.05 were deemed meaningful and subjected to post-hoc testing using the Holm-Bonferroni method to control for family-wise error rate resulting from multiple pairwise comparisons. To this end, the EMMEANS package was used to extract and compare estimated marginal means, and thereby take account of the partially crossed design. The function rating.emmeans() function (see RVAideMemoire, v0.9-81-2) was also used to get the probability of each rating score. To analyse the measures of gaze location, linear mixed effects modelling with an identity link function was also used but with Viewing Condition as a fixed effect for 3D ellipse volume, and AOI and Viewing Condition as fixed effects for gaze location.

## 3 Results

### 3.1 Performance data

**Figure 3:**
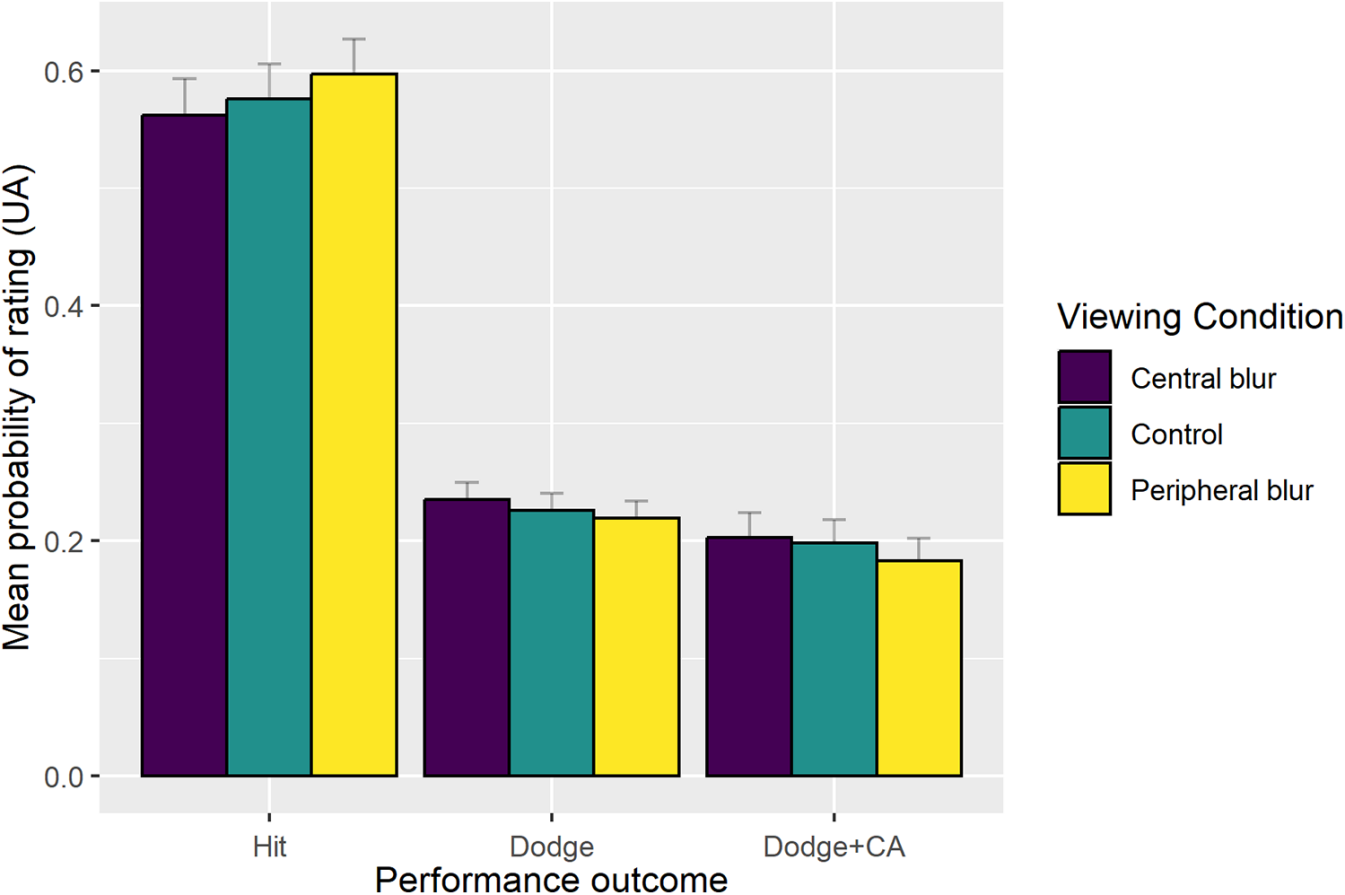
Group mean (+SE) probability of performance outcome (hit, dodge, counterattack—CA) as a function of sequence type.

The final reduced model (AIC = 3795.597, conditional *R*^2^ = 0.066) indicated a main effect of Sequence Type [*χ*^2^ (1) = 21.44, *p* < .0001]. Performance was better when participants faced sequences of one punch compared to sequences of two punches. There were similar mean probabilities of a dodge (0.255) and counterattack (0.244) when facing one punch sequences. However, when facing two punch sequences there was a reduced probability of a counterattack (0.145) compared to a dodge (0.198). As can be seen in Figure 3, there was no significant effect of Viewing condition. In the central blur condition, the mean probability of a dodge was 0.236 and 0.200 for a counterattack, whereas the mean probability of a dodge was 0.222 and 0.177 for a counterattack in the peripheral blur condition. In the control condition, the mean probability of a dodge was 0.230 and 0.190 for a counterattack.

For the distance between the participant and the opponent, the full model (AIC = −7.990, conditional *R*^2^ = 0.81) indicated a significant interaction between Sequence Type and Viewing Condition [*χ*^2^ (2) = 6.929, *p* = .03]. No significant main effects of Viewing Condition or Sequence Type were found. Post-hoc testing of the interaction did not indicate significant differences between relevant comparisons. Observation of mean data indicated that the participant-opponent distance tended to be smaller in peripheral blur condition, especially when facing two punches sequences (1.27 m), compared to the control (1.36 m) and central (1.47 m) conditions. Overall, the mean distance was 1.36 m.

### 3.2 Gaze location

For 3D ellipse volume across the entire block duration, the reduced model (AIC = 36.935; conditional *R*^2^ = 0.73) indicated a significant main effect of Viewing Condition [*χ* (2) = 7.254, *p* = .02]. However, post-hoc testing did not identify any significant differences. The 3D ellipse volume was 96 cm^3^ in the control condition, 96.5 cm^3^ in the central blur condition and 121.8 cm^3^ in the peripheral blur condition (*p* = .090 when comparing central blur to peripheral blur and control to peripheral blur; *p* = 0.96 when comparing central blur to control condition).

**Figure 4:**
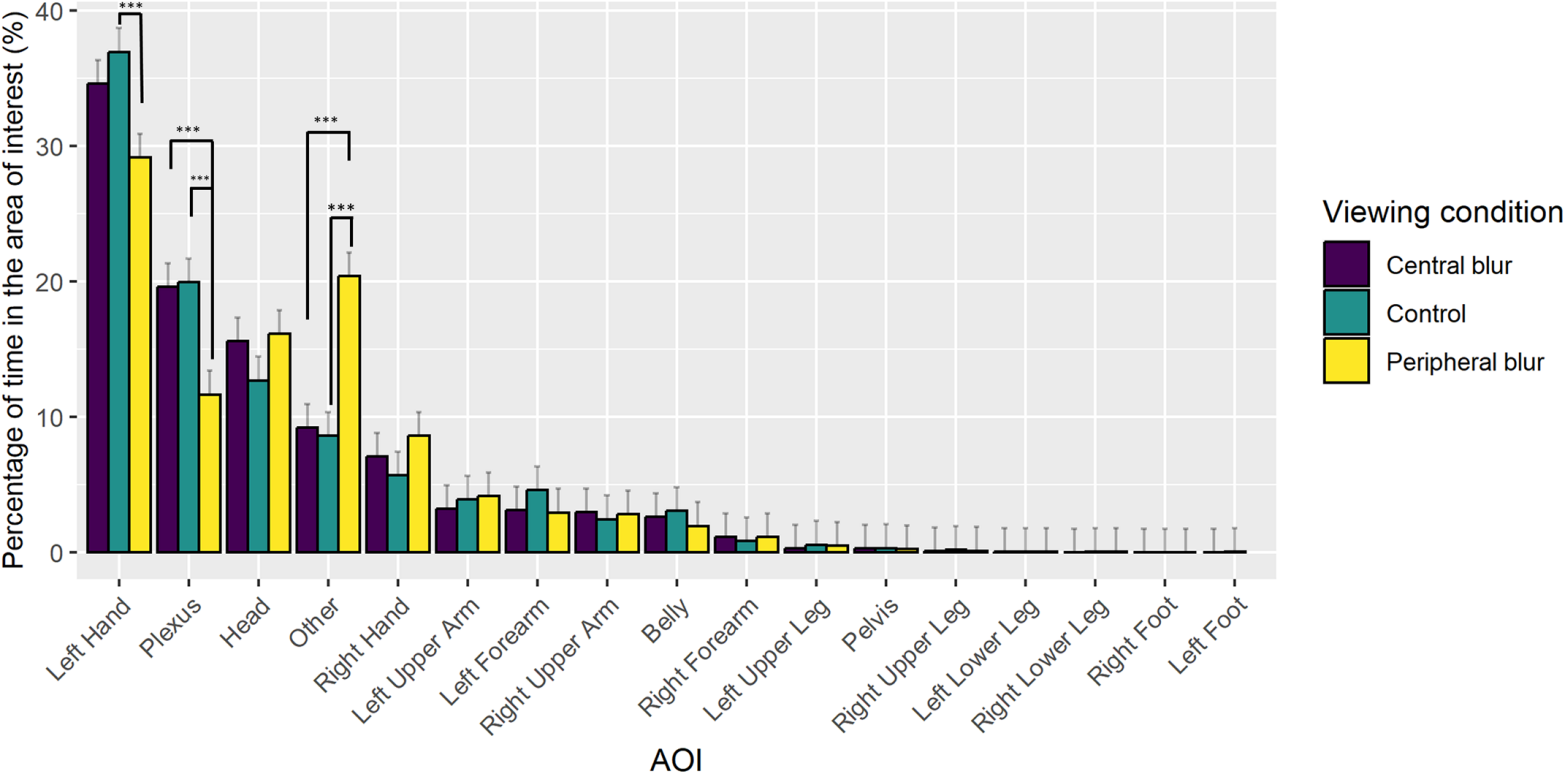
Group mean (+SE) percentage of time that gaze was located in the AOIs as a function of viewing condition. Significant pairwise comparisons indicated with ***.

For the analysis of gaze location with respect to AOIs over the entire block, the reduced model (AIC = 2,773.832, conditional *R*^2^ = 0.84) indicated a significant main effect of AOI [*χ*^2^ (16) = 2,582.860, *p* < 0.0001], as well as a significant interaction between AOI and Viewing Condition [*χ*^2^ (32) = 117.410, *p* < 0.0001]. As can be seen in Figure 4, participants spent a lower percentage of time with gaze located on the left hand and the plexus in favour of areas other than the boxer in peripheral blur condition compared to the central blur and control conditions. Consistent with the larger 3D ellipse volume, the location of the gaze away from the boxer increased in the peripheral blur condition (20.29%) compared to the control condition (8.63, *p* < 0.0001) and the central blur condition (9.20, *p* < 0.0001).

## 4 Discussion

Like athletes in many sports that involve interceptive and/or avoidance actions, boxers use visual information to anticipate and respond to an upcoming event, such as opponent's attacking punch (2, 3). Therefore, it is relevant to develop greater understanding of the visual processes that underpin these boxing behaviours and whether they are modified by targeted manipulations of the visual surrounds. Motivated by limitations of *in situ* studies on the impact of dioptric blur in cricket batting (24, 25), and lab-based blur manipulations of 2D stimuli in tennis (14), here we used an immersive and adaptive VR protocol to investigate the effect of a gaze-contingent blur in a group of elite boxers. Importantly, to better reflect the demands of a realistic boxing encounter with an opponent, we adapted the task used in our recent study (18) such that participants were required to dodge one or two attacking punches from the opponent (20), and then make a counterattack.

In this more representative VR boxing task, we found that elite boxers were able to maintain outcome performance when a gaze-contingent blur was introduced to the central or peripheral visual field (18). Resilience to the introduction of blur was also evident in the distance between the participant and the opponent, which did not differ significantly between any of the viewing conditions. Elite boxers did, however, exhibit better outcome performance when they faced sequences of one punch than two punches. Irrespective of the presence of blur, elite boxers were able to anticipate reasonably well a single punch (using visual cues from the opponent's movement), thereby giving them time to avoid being hit and to make a counterattack, but not if there was a subsequent second punch. Observation of the mean data indicated that elite boxers exhibited a similar probability of dodging (0.255) and dodging plus counterattacking (0.244) when facing one punch sequences. When facing two punch sequences there was a reduced probability of dodging plus counterattacking (0.145) compared to dodging (0.198). In other words, when elite boxers faced two-punch sequences, they found it more difficult to dodge and then counterattack the virtual opponent.

The 3D ellipse data indicated a broader spread of gaze location in the peripheral blur condition, which was confirmed by the analysis of gaze location with respect to AOIs. In the peripheral blur condition, the percentage time with gaze located on the left hand and the plexus was reduced in favour areas other than the boxer, compared to the central blur and control conditions. Given that low-spatial frequency information on motion and/or the spatial surrounds would have been available in the peripheral blur condition, it is not immediately obvious why participants gazed more often at areas other than the opponent. However, as first commented in Limballe et al. (29), our use of a Gaussian blur did not include a blur gradient that changed as a function of depth, and did not mimic identically the physiological optics of the retina [see (29)]. This may have impacted upon participants' perception of spatial orientation, leading them to shift gaze away from the opponent in order to confirm their location in the ring relative to the surrounds. In the central blur condition, participants spent a greater percentage of time with gaze located on the left hand and plexus, which is broadly in line with the relatively concentrated location of gaze, and hence overt attention, on upper body and head observed in French boxing (30) and karate (6), as well as in our previous study of VR boxing (18). Interestingly, however, the difference in gaze location between the peripheral and central blur conditions did not lead to differences in outcome performance.

Extending upon our study of expert combat athletes performing a chasing-parry like task (18), the current study examined elite boxers with the expectation that they would have sufficient skill to dodge and then counterattack when faced with 1 or 2-punch attacking sequences from a realistic opponent (i.e., also an elite boxer). We did not consider if there are skill-level differences in participants' response to the blur manipulation, but previous work has shown that novice through to expert level athletes in a range of sports are relatively impervious to the presence of blur (14, 18, 25, 31, 32). There is also some evidence of a facilitatory effect on performance of novices when they are provided with clear central vision and blurred peripheral vision (16). It has been suggested that resilience to blur could be a result of participants not requiring high spatial frequency visual information (25), or that they shift their reliance from using high to low spatial frequency visual information (14, 16, 19, 33), or proprioception (24). Whether performance in elite boxers can be improved by targeted blur manipulations in a VR setting remains to be determined, but in this respect it is interesting to hear anecdotal reports from boxers and coaches in the current study who suggested that during a fight, vision deteriorates with fatigue and repetitive blows to the head. Thus, it could be the case that boxers might benefit from safely practicing in a blurred vision condition if this provides an opportunity to familiarise to this specific situation. Related, a primary concern for boxers and their coaches is the management of injury (e.g., traumatic or mild concussive) and return to combat. VR can offer a boxer the opportunity to recover from injury while still being able to practice in a setting that presents realistic information on the opponent. However, it is important that the VR environment represents the temporal and spatial constraints of typical attacking sequences (i.e., dodge and counterattack). This was an important aim of the current study, and as such it is relevant to note that the feeling of presence in the VR environment was strong, with participants commenting that they “felt” the ring and experienced a realistic information intake. An implication is that the type of VR stimuli used in the current study could help injured boxers reduce feelings of missing the opportunity to be in the ring and interact with an opponent (2). That said, some participants in the current study commented that the essential point of boxing was missing because there was no danger and no impact from taking and/or giving a punch. The implication is that practice in an immersive and realistic virtual boxing environment, including targeted visual manipulations, could be used to complement but not replace traditional boxing training methods.

Here, it is important to recognize that although eye tracking within an HMD did enable a gaze-contingent manipulation within an immersive and realistic boxing task [for an example of gaze-contingent simulation of clinical visual impairments see (34)], there are some considerations. First, the spatial and temporal resolution of the eye tracker may not have been sufficient to identify small amplitude eye movements, meaning that there may have been instances where the gaze-contingent blur was not aligned with gaze location. Any misalignment would likely have been small and of short duration, such as if the eye tracker did not register a 1 deg return saccade. Second, it is possible that there could have been some headset slippage while performing the task, which would have influenced the location of the gaze-contingent manipulation, as well as the time spent with gaze located within each AOI. To minimise headset slippage, we made efforts to ensure that the HMD was well-fitted (NB. HTC VIVE EYE PRO incorporates a face mark and two fastening straps) and that the eye tracker was calibrated prior to each block of 60 trials. Having done so, at no point during or after the testing session did participants comment about headset slippage, or a misalignment between gaze and the blur manipulation. Also, although we made no attempt to quantify headset slippage on gaze accuracy [for effects of slippage while wearing eye tracker glasses with a head strap as a function of different movement tasks see (35)], if it did occur, we suspect that it was probably quite small given that the distribution of gaze within the AOIs was consistent with previous work (3, 4). That said, to ensure accuracy of gaze tracking in future work it might be prudent to determine gaze offset during and/or after the end of each block of experimental trials by asking participants to fixate gaze on known locations. A third issue concerns our analysis of eye gaze, which did not consider measures such as fixation rate, fixation duration and scan path. Previous work has used a dispersion algorithm to determine measures of fixation when standing stationary and shifting gaze between several static targets in a VR environment (36). However, it remains unclear if this approach is suitable when there is rapid head translation and rotation, such as in VR boxing. A dedicated validation study will be needed to answer this question. Future work could also consider the effectiveness of algorithms that classify fixations based on identifying saccades. For example, although saccade identification based on velocity thresholds can be subject to error within eye data recorded at low sampling rates [e.g., <60 Hz; (37)], improvements can be made through careful consideration of signal processing such as filtering and interpolation (38). More recently, Gibaldi and Sabatini (39), have reported that modelling eye position during a saccade (i.e., saccade trajectory) as a Sigmoid, enables reliable identification of saccade amplitude, duration and peak velocity with sampling rates as low as 50 Hz. Finally, it is relevant to comment that our decision to analyse gaze with respect to 17AOIs [see also (5)] could have resulted in some misclassification, such as when gaze was located near the border of two connected AOIs (e.g., hand and forearm). This is a problem common to eye tracking studies, and may potentially be reduced by using fewer and more spatially separated AOIs. In combat sports, there are previous studies that classified gaze location with respect to 6 AOIs (3, 30). While this has the potential to result in fewer misclassifications (e.g., no distinction between lower and upper leg), it is typically a subjective process done by the experimenter during post-processing. As such, there is still the possibility of making errors when classifying fixation on AOIs that are in close proximity and not easily distinguishable (e.g., upper torso vs. lower torso, or legs vs. hips). To minimize subjective classification errors, we followed an objective process using functions available from a dedicated software development kit (SDK) in Unity software. This resulted in gaze being located on 5 unconnected AOIs that were not in close proximity (left hand, plexus, head, other and right hand) for the majority of time. In the remaining 12 AOIs, there was no influence of Viewing Condition, leading us to suggest that any misclassification in a small percentage of the data would not have impacted upon the findings.

## 5 Conclusion

This study contributes to understanding of how a gaze-contingent blur impacts upon on dodging and counterattack performance, as well as eye gaze during a realistic VR boxing task with an elite population. A resilience to the presence of blur, accompanied by a change in how gaze was located within the dynamic visual scene, is indicative of a flexible use of the information available. In future work, it will be relevant to consider whether VR training with a gaze-contingent blur can facilitate learning and transfer to a real boxing situation.

## Data Availability

The raw data supporting the conclusions of this article will be made available by the authors, without undue reservation.
