# Supplementary material for: Virtual reality boxing: impact of gaze-contingent blur on elite boxers performance and gaze behavior
Source: Front Sports Act Living. 2024 Dec 24;6:1430719. doi: 10.3389/fspor.2024.1430719 (PMC11703828; doi:10.3389/fspor.2024.1430719)
Supplement: Supplementary file 1 [file Table1.docx]

Table 1: Description of punches with the 20 unique attacking sequences

| Sequence Number | Number of punches | Description |
| --- | --- | --- |
| 1 | 1 | Left straight to the head |
| 2 | 1 | Right cross to the head |
| 3 | 1 | Right straight to the head |
| 4 | 1 | Left straight to the head |
| 5 | 1 | Left straight to the head |
| 6 | 1 | Right straight to the head |
| 7 | 1 | Left cross to the head |
| 8 | 1 | Left cross to the body |
| 9 | 1 | Right cross to the head |
| 10 | 1 | Right straight to the head |
| 11 | 2 | Left straight - right straight to the head |
| 12 | 2 | Left straight - left straight to the head |
| 13 | 2 | Right straight - right straight to the head |
| 14 | 2 | Left straight - right cross to the head |
| 15 | 2 | Right straight - left cross to the head |
| 16 | 2 | Right cross - left straight to the head |
| 17 | 2 | Left straight - right cross to the head |
| 18 | 2 | Right cross to the head - left cross to the body |
| 19 | 2 | Right cross to the body - left cross to the head |
| 20 | 2 | Right straight - left cross to the head (exit left) |
